# How should the respiration rate be counted in cattle?

**DOI:** 10.1007/s11259-022-09984-7

**Published:** 2022-08-17

**Authors:** L. Dißmann, J. Heinicke, K. C. Jensen, T. Amon, G. Hoffmann

**Affiliations:** 1 Department of Engineering for Livestock Management, Leibniz Institute for Agricultural Engineering and Bioeconomy (ATB), Max-Eyth-Allee 100, 14469 Potsdam, Germany; 2 Institute for Veterinary Epidemiology and Biostatistics, Department of Veterinary Medicine, Freie Universität Berlin, Königsweg 67, Building 21, 14163 Berlin, Germany; 3 Institute of Animal Hygiene and Environmental Health, Department of Veterinary Medicine, Freie Universität Berlin, Robert-von-Ostertag-Str. 7-13, 14163 Berlin, Germany

**Keywords:** Respiration rate, Method, Respiration rate sensor, Flank movement

## Abstract

Respiration rate (RR) is a proficient indicator to measure the health status of cattle. The common method of measurement is to count the number of respiratory cycles each minute based on flank movements. However, there is no consistent method of execution. In previous studies, various methods have been described, including counting flank movements for 15 s, 30 s or 60 s as well as stopping the time for 5 or 10 breaths. We assume that the accuracy of the aforementioned methods differs. Therefore, we compared their precision with an RR sensor, which was used as the reference method in this study. Five scientists from the fields of agricultural science and veterinary medicine quantified the flank movement according to each of the five methods mentioned above. The results showed that with an average RR of 30 breaths per minute (bpm), all methods showed a high correlation to the values of the RR sensor. However, counting breaths for 60 s had the highest level of conformity with the RR sensor (Lin`s concordance correlation coefficient: 0.96) regardless of the level of RR. With rising RR, the inaccuracy increased significantly for the other four investigated methods, especially when counting 5 and 10 breaths. Therefore, we would recommend that counting for 60 s should be used as the standard method for future studies due to its high precision regardless of the level of RR.

## Introduction

Respiration rate (RR) is an important parameter to evaluate the health status of cattle since it is an indicator of stress and painful processes (Knickel et al. 2000;  Rosenberger 1990) as well as heat exposure (Pinto et al. 2019; Schütz et al. 2014). There are several approaches to automatically record the RR. Some of the most innovative methods are infrared thermography (IRT) techniques based on measuring the temperature change of the inhaled and exhaled airflow through the nostrils (Jorquera-Chavez et al. 2019; Kim and Hidaka 2021; Lowe et al. 2019). Additionally, there are sensor systems such as a differential pressure sensor fixed on the nose (Strutzke et al. 2019), a respiration monitoring system strapped to the cow`s abdomen as a belt (Eigenberg et al. 2000), a laser distance sensor recording the body movement of the regio abdominis (Pastell et al. 2007) and a sensor measuring air temperature near the nostrils (Milan et al. 2016).

Nevertheless, observing flank movements is still the most common technique in practice to measure the RR and is often used as a reference method for validation of the sensor systems mentioned above. However, there is no clear gold standard in cattle, and we found various methods described in scientific papers. One method is to measure the time until 5 breaths are completed, applied by Kim and Hidaka (2021) and Lowe et al. (2019) as a reference method to analyze the breathing pattern with IRT. Lowe et al. (2019) argues that this method reduces the probability of calf movements during counting.

Another frequent technique is to measure the time until 10 breaths are fulfilled. This method was employed by Stewart et al. (2017) as a reference value for their infrared-based measurements, Schütz et al. (2014) in their study on the influence of different amounts of shade on the RR of cattle in relation to heat load and Li et al. (2020) with regard to the correlation between respiration and rectal temperature as well as the prediction of the RR.

In other studies, the RR measurement was accomplished through a time limit instead of a breath limit. Maia et al. (2014) counted flank movements for 15 s after putting a face mask on cattle to measure the physiological response. The textbook Clinical Propaedeutics of Domestic Animals (Baumgartner et al. 2018) recommends counting at least 30 s of flank movement, also applied by Pinto et al. (2019) in their examination of the influence of climate and circumstantial factors on RR in cows. Jorquera-Chavez et al. (2019), Milan et al. (2016) and Strutzke et al. (2019) counted the RR for 60 s as a reference method for validating their sensor systems.

In the present study, we tested and compared the various methods described (counting 5 and 10 breaths, 15 s, 30 s and 60 s) regarding their agreement with respect to the measured RR of an RR sensor, recently developed in our working group (Strutzke et al. 2019) and used as the reference method in this study. This sensor automatically calculates the RR in the nose during inspiration and expiration. We hypothesize that shorter periods of observation increase inaccuracy. Our aim is to define the most meticulous method for counting RR that should be consistently used in further studies.

## Materials and methods

In total, 46 episodes were recorded (Samsung Galaxy Note 10.1, Seoul, South Korea) over one minute each in an experimental barn in Groß Kreutz (Germany) over 2 days in January 2018. In the experiment, six healthy cows differing in age, lactation stage (1^st^-5^th^ lactation, days in milk: 47–196) and gestation stage (4–80 days) were filmed consistently at a 45-degree angle from behind. The dairy cows were housed in a free-stall barn, equipped with 53 lying cubicles (straw-lime mixture) and were part of an existing herd of 55 cows on the first day and 54 cows on the second day. The animals were able to move freely in the barn during the experiment so as not to restrict their natural behavior. Water and a total mixed ration were freely available. Seven to nine video sequences were taken of each cow during the lying and standing periods (60% lying, 40% standing), with and without ruminating and while dozing (eyes half-closed, without rumination). In addition, each cow was equipped with a respiration rate (RR) sensor attached to a halter (Strutzke et al. 2019). The experimental study using animals was approved by the State Office for Occupational Safety, Consumer Protection and Health (LAVG Brandenburg, Germany) under the study number 2340–1-2018.

Afterward, three veterinarians and two agricultural scientists counted the RR on the basis of the video sequences according to the five methods (5 breaths, 10 breaths, 15 s, 30 s and 60 s) in random order using a smartphone stopwatch app. By means of an LED lamp that lights up briefly at the beginning and end of the recorded minute, the period to be studied was dependably defined. The LED lamps were fixed on the halter of the cows and were synchronized with a marker in the RR sensor recordings (for further details see Strutzke et al. 2019). Only whole breaths were counted, beginning with the inspiration after the first light of each video minute. When counting by breaths, the time was recorded in seconds with two decimals. Values were then extrapolated to breaths per minute (bpm) and compared to the value of the RR sensor.

We compared the five methods regarding their validity (agreement of the measurement results with the RR sensor) and reliability (differences between observers). The number of cases “n” was obtained from the 46 studied videos and multiplied by five (number of observers). First, we determined the mean absolute deviation of the studied methods compared to the RR of the sensor and investigated whether there were differences depending on the level of RR. For this, we used JMP (Version 16.0, SAS Institute Inc., Cary, NC, USA). To assess the agreement between the five different methods and the RR sensor, Lin's concordance correlation coefficient (CCC) (Akoglu 2018) was calculated. To detect differences regarding the reliability, we additionally calculated the CCC for each observer. For the CCC, we used R via the R Studio Interface (Version 4.0.3.; © 2020, The R Foundation for Statistical Computing) and the package “DescTools” (Signorell et al. 2021). Lin’s CCC is particularly suitable to measure the agreement of two methods or raters, as it is based on precision (degree of variation) and accuracy (degree of location or scale shift) (Barnhart et al. 2002).

## Results and discussion

**Fig. 1:**
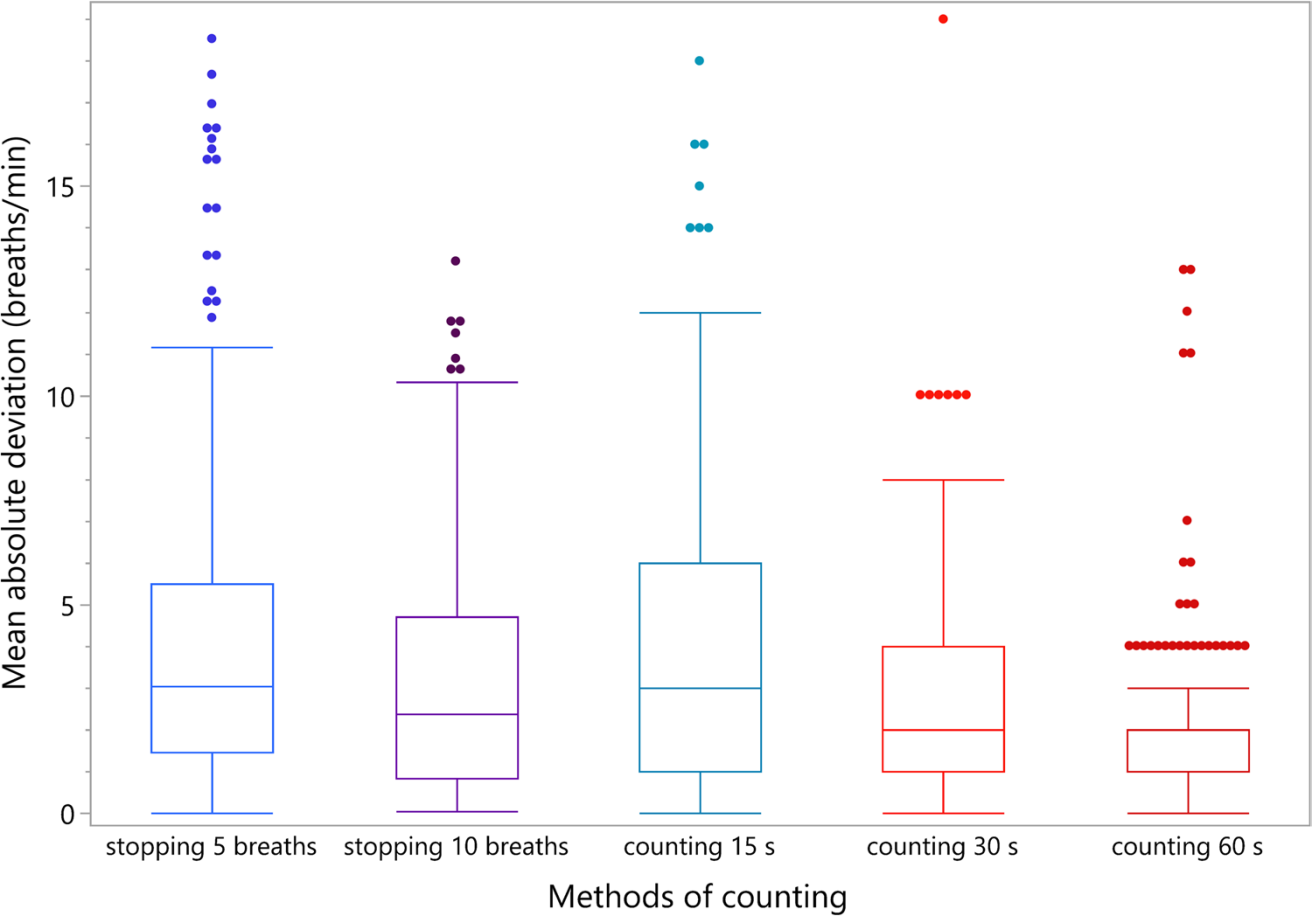
Boxplot analysis of the mean absolute deviation of the respiration rate (RR) from the RR sensor by the five investigated methods of counting (n = 230)

**Fig. 2:**
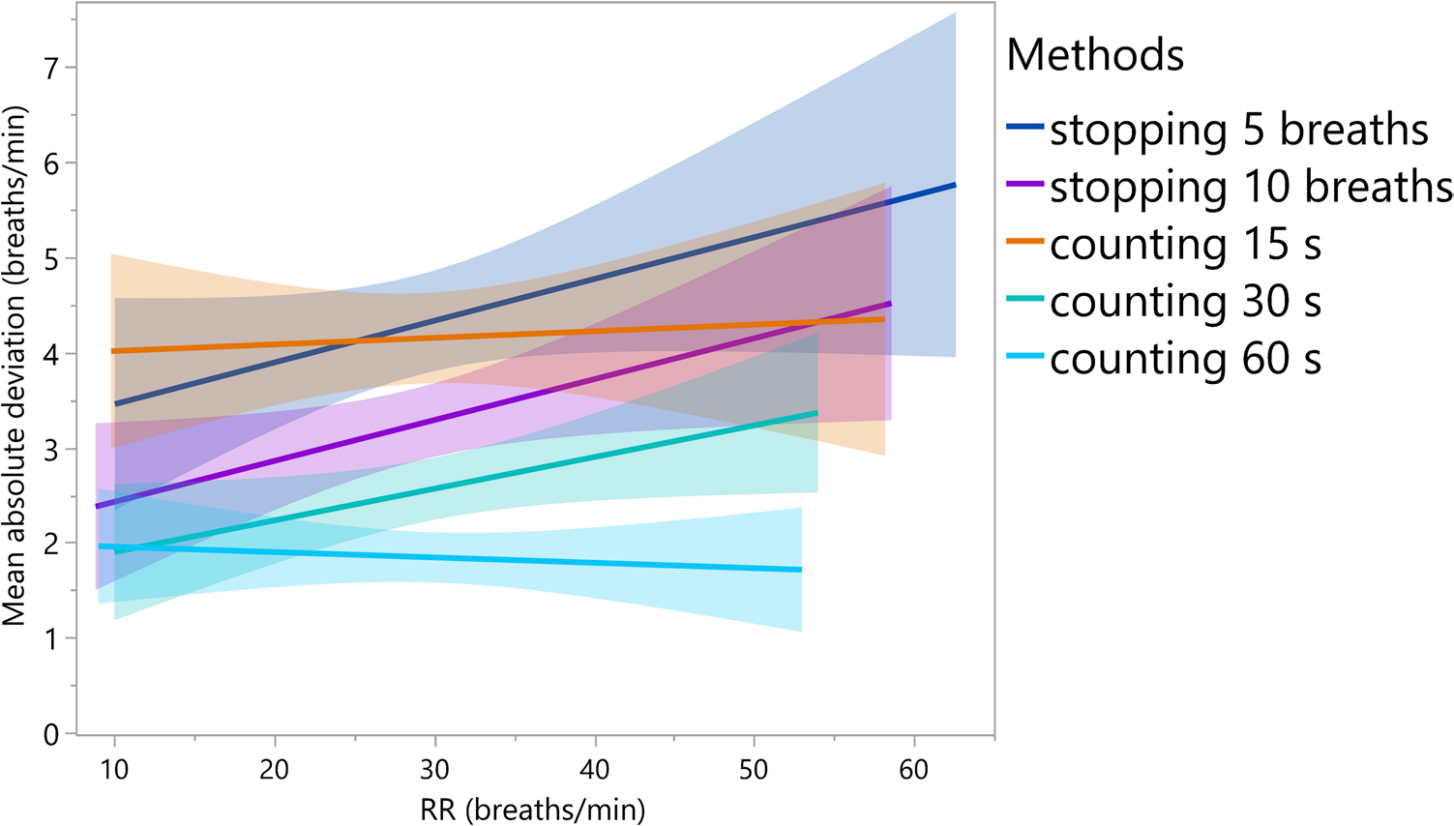
Mean absolute deviation of the five investigated methods for the respiration rate (RR) sensor against the level of RR (breaths/min) from 46 recordings counted by five observers (n = 230)

The results substantiate that counting respiration rate (RR) for 60 s has the lowest mean absolute deviation and therefore the highest level of agreement with the values measured by the RR sensor regardless of the level of RR. Counting for 60 s differed from the RR sensor by an average of 1.8 breaths with a standard deviation of 2.02. The second highest level of agreement can be attributed to counting breaths for 30 s, followed by stopping for 10 breaths (Fig. 1). Up to an RR of 25 bpm, counting 5 breaths was more accurate than counting breaths for 15 s. At a higher RR, counting breaths for 15 s became more accurate than counting 5 breaths. Overall, the deviation from the RR sensor was on average smaller at a low RR and increased with rising RR for all methods, except for counting for 60 s. At 60 s, the deviation remained approximately constant regardless of the RR (Fig. 2).

In our experiment, the average RR was 30 bpm; thus, counting 5 breaths corresponded to an average observation time of 10 s, and counting 10 breaths corresponded to an average observation time of 20 s.

Moreover, it was noteworthy that for all methods, the RR was on average underestimated at a low RR and overestimated at a high RR. However, the position of the cow (lying or standing) had no influence on the detectability of the RR, and the mean absolute deviation from the RR sensor was approximately the same for both positions.

Regarding the CCC, all methods achieved a CCC > 0.8 (Table 1). However, there were differences concerning the single methods: the level of agreement of the investigated methods proved to be in the same order as with regard to the mean absolute deviation. There are different approaches for the interpretation of Lin´s CCC (Akoglu 2018): According to McBride (2005), only the counting of breaths for 60 s achieved a substantial agreement (0.95–0.99).

**Table 1:** Agreement of different counting methods for the respiration rate (RR) with the measurement of an RR sensor using Lin´s concordance correlation coefficient (CCC) for all observers (n = 230) and for the best and worst of five observers (n = 46)

Method	All observers CCC (LCI-UCI)	Best observer CCC (LCI-UCI)	Worst observer CCC (LCI-UCI)
Counting RR for 15 s	0.84 (0.80–0.88)	0.87 (0.79–0.93)	0.79 (0.66–0.87)
Counting RR for 30 s	0.93 (0.91–0.95)	0.95 (0.91–0.97)	0.90 (0.83–0.94)
Counting RR for 60 s	0.96 (0.95–0.97)	0.97 (0.94–0.98)	0.95 (0.92–0.97)
Stopping time for 5 breaths	0.82 (0.77–0.86)	0.88 (0.80–0.93)	0.78 (0.64–0.87)
Stopping time for 10 breaths	0.90 (0.87–0.92)	0.93 (0.87–0.96)	0.86 (0.77–0.92)

LCI: lower confidence interval, UCI: upper confidence interval

Concerning the reliability, the five observers differed only slightly when comparing the CCC of the different methods (Table 1). All five observers reached a substantial agreement with the RR when counting breaths for 60 s. Therefore, we conclude that the reliability of this method is sufficient.

Overall, the hypothesis that longer observation times result in a more accurate RR measurement was confirmed. Therefore, we would generally recommend using counting for 60 s as the standard method in future studies because it is the most accurate method regardless of the level of RR. An exception are very restless animals, where a longer observation period would distort the results due to cow movements and make counting flank movement more difficult, for example, in calves (Lowe et al. 2019).

Although the average RR in cattle is between 24 and 36 bpm (Rosenberger 1990), RRs of 78 bpm are not unusual in summer (Ruban et al. 2020). At these high RRs, we consider counting 5 or 10 breaths to be too inaccurate due to their short observation time. The accuracy of counting for 15 s and 30 s deteriorated less in our experiment at higher RRs than counting by breaths (Fig. 2). Consequently, for a basic acquisition of RR in daily work in practice, counting breaths for 30 s can be a good alternative to counting for 60 s considering validity, reliability and feasibility (less work).

Nevertheless, when counting 5 and 10 breaths, it is necessary to consider the reaction time in counting the last breath and stopping the stopwatch of the person evaluating the video. Even with rigorously trained researchers, human physical limitations will inevitably influence the study results. Furthermore, when extrapolating up to one minute, the number of breaths must be rounded down as well as up to obtain whole breaths.

In fact, when counting by time, different initial conditions must be considered; when counting for 15 s and then rounding up to one minute by multiplying by 4, logically from the outset, only every fourth value can be obtained as an end result, and when counting for 30 s, only every second value can be obtained. Therefore, we conclude that the counting of breaths for 60 s is the most valid method.

## Data Availability

The data that support the findings of this study are not openly available due to reasons of sensitivity but are available from the corresponding author upon reasonable request.
